# Supplementary material for: Insights into the Gut Microbiome of the South American Leaf-Toed Gecko (Phylodactylus gerropygus) Inhabiting the Core of the Atacama Desert
Source: Microorganisms. 2024 Jun 13;12(6):1194. doi: 10.3390/microorganisms12061194 (PMC11205927; doi:10.3390/microorganisms12061194)

Insights into the gut microbiome of the South American leaf-toed gecko (*Phylodactylus gerropygus*) inhabiting the core of the Atacama Desert

Daniela S. Rivera[a], Valentina Beltrán[a], Ignacio Gutiérrez-Cortés[a,b], Constanza Vargas[c] & Fernando D. Alfaro[a]

[a] GEMA Center for Genomics, Ecology & Environment, Universidad Mayor, Camino La Piramide, 5750, Huechuraba, Santiago, Chile.

[b] Extreme Ecosystem Microbiomics & Ecogenomics Lab., Facultad de Ciencias Biológicas, Pontificia Universidad Católica de Chile, Santiago, Chile.

[c] Centro UC Desierto de Atacama, Pontificia Universidad Católica de Chile, Alameda 340, Santiago, Chile.

Current address: Daniela S. Rivera, GEMA Center for Genomics, Ecology & Environment, Universidad Mayor, Camino La Piramide, 5750, Huechuraba, Santiago, Chile.

Email: daniela.rivera@umayor.cl

Short title: Gut microbiome of the leaf-toed gecko

Keywords: gut microbiome, *Phyllodactylus gerropygus*, Atacama Desert

Supplementary material

Figure S1. Diversity and gut microbiome richness in wild-caught South American leaf-toed geckos (*Phyllodactylus gerropygus*). Rarefaction curves comparing the number of observed ASVs found in female and male geckos.

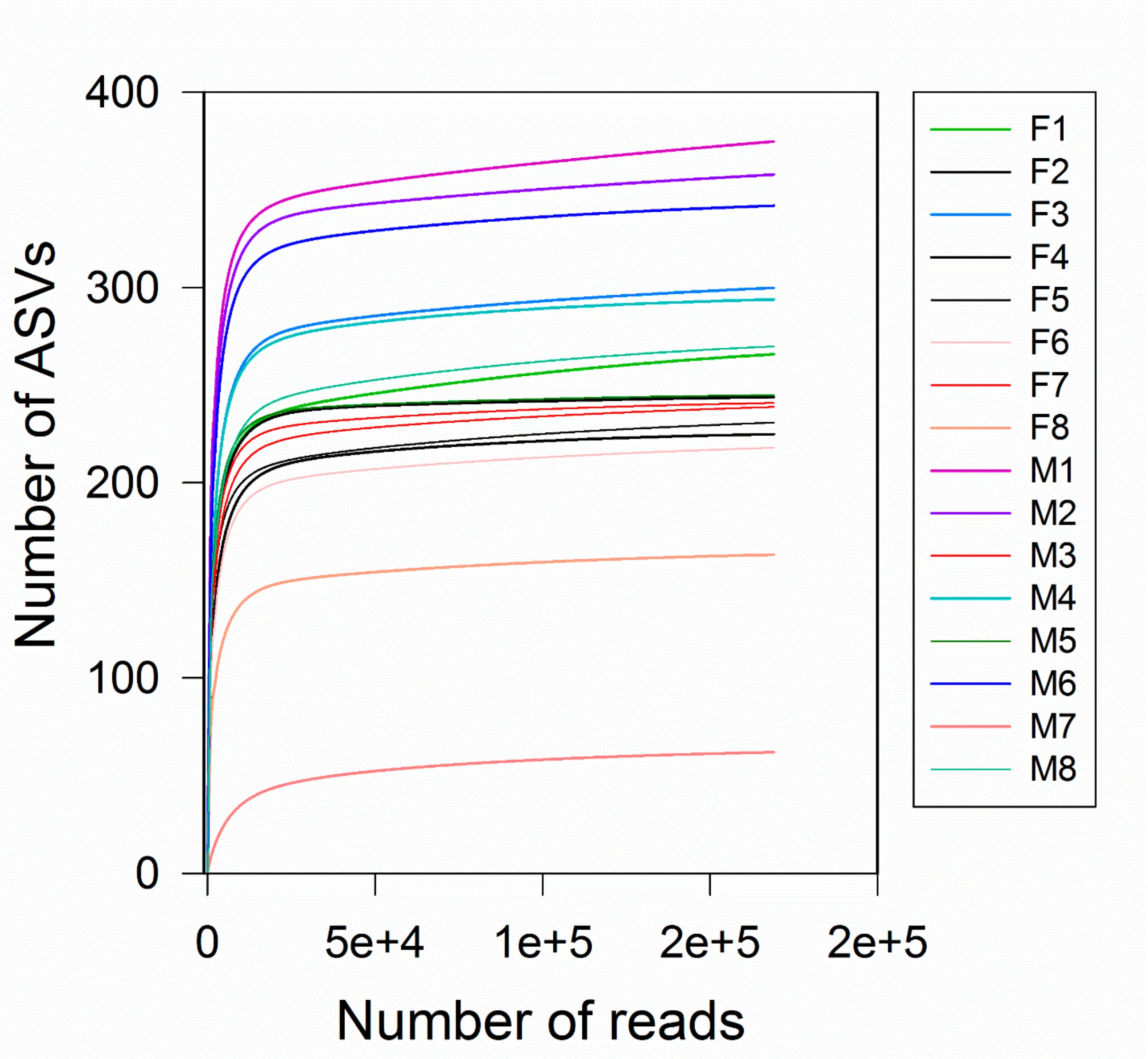

Supplement: Supplementary file 1 [file microorganisms-12-01194-s001.zip › microorganisms-2999538-supplementary.pdf]
